# Supplementary material for: Revisiting the Last Ice Area projections from a high-resolution Global Earth System Model
Source: Commun Earth Environ. 2025 Jan 23;6(1):46. doi: 10.1038/s43247-025-02034-5 (PMC11754091; doi:10.1038/s43247-025-02034-5)
Supplement: Supplementary file 2 — Supplementary material—Revisiting the Last Ice Area projections from a high-resolution Global Earth System Model [file 43247_2025_2034_MOESM2_ESM.pdf]

Supplementary information - Revisiting the Last Ice Area projections from a high-resolution Global Earth System Model

Madeleine Fol[1], Bruno Tremblay[1-2], Stephanie Pfirman[3], Robert Newton[2], Stephen Howell[4], Jean-François Lemieux[5]

1 Department of Atmospheric and Oceanic Sciences, McGill University, Montréal, QC, Canada

2 Lamont-Doherty Earth Observatory, Columbia University, Palisades, NY, USA

3 College of Global Futures, Arizona State University, Tempe, AZ, USA

4 Climate Research Division, Environment and Climate Change Canada, Toronto, ON, Canada

5 Recherche en Prévision Numérique Environnementale/Environnement et Changement Climatique Canada, Dorval, QC, Canada

Supplementary figures

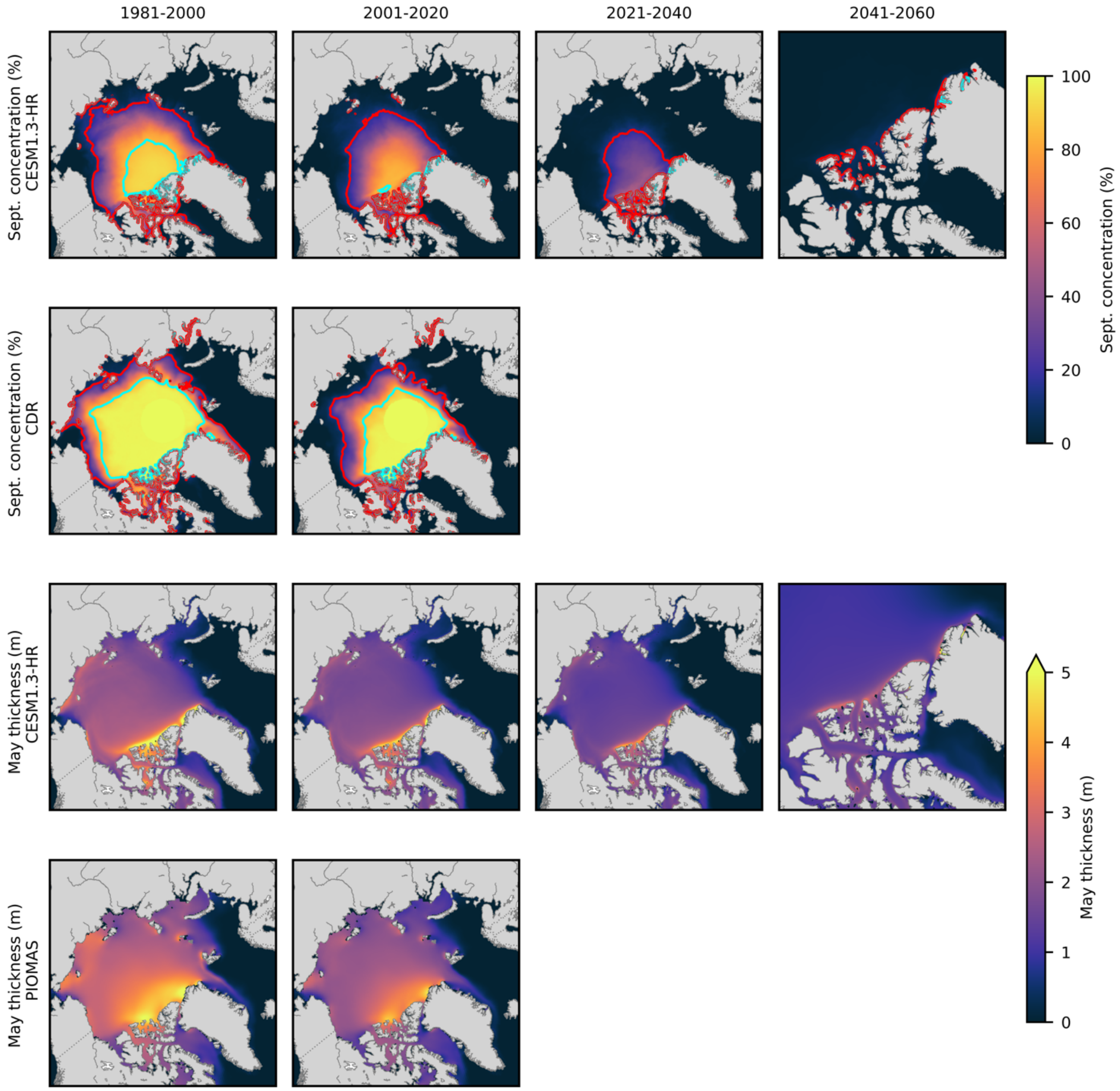

Fig. S1. Observed (CDR) and simulated (PIOMAS, CESM1.3-HR) ensemble mean maximum September sea ice concentration and minimum May sea ice thickness for the period 1981-2000, 2001-2020, 2021-2040, and 2041-2060. The simulated and observed marginal ice zone (15% and 85% SIC) contours are shown in red and cyan.

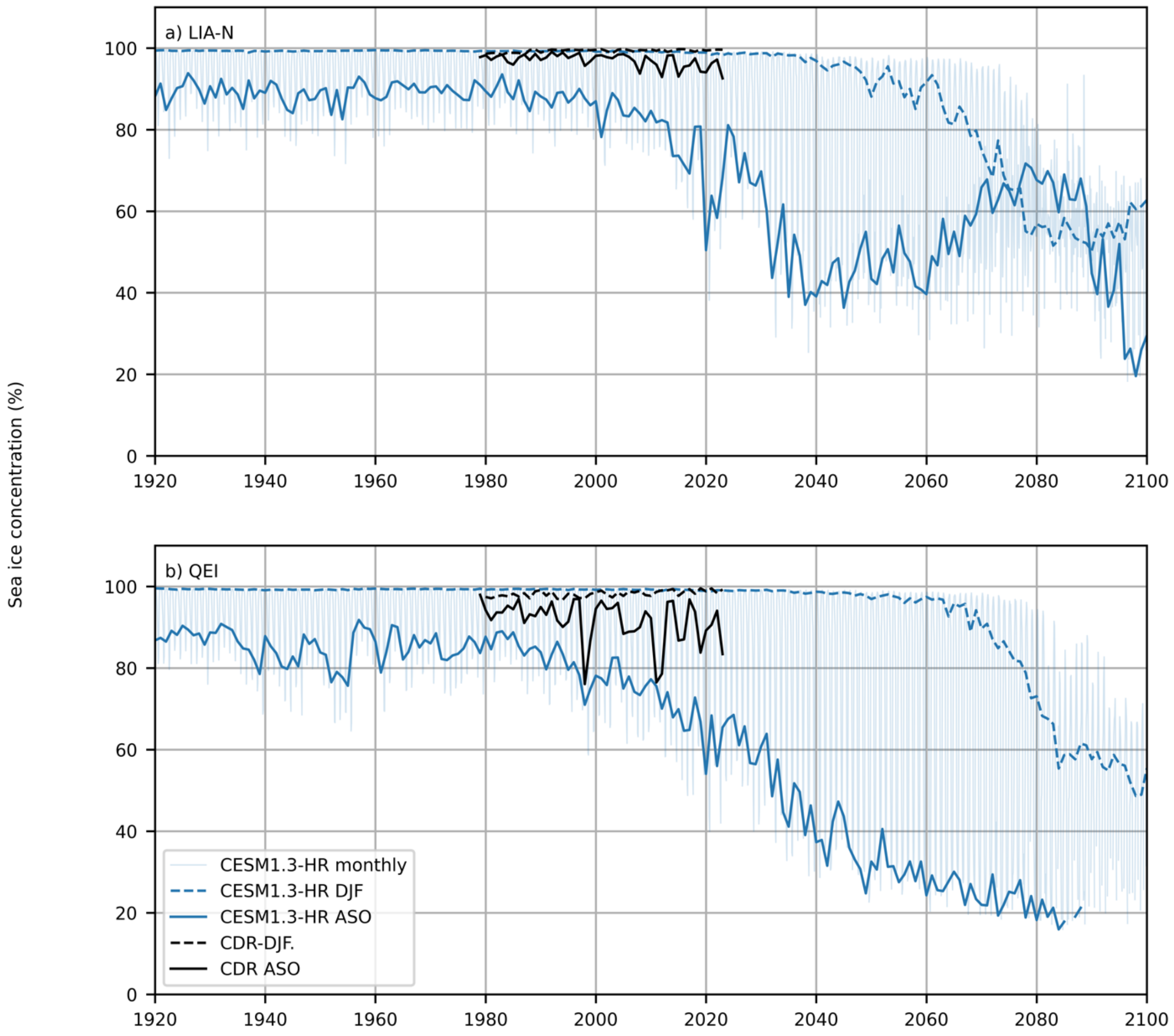

Fig. S2. Observed and simulated December-January-February (dashed lines) and August-September-October (full lines) mean sea ice concentration for CESM1.3-HR (blue) and CDR (black) in the LIA-N (a) and the QEI (b). The simulated monthly mean time series is shown in light blue.

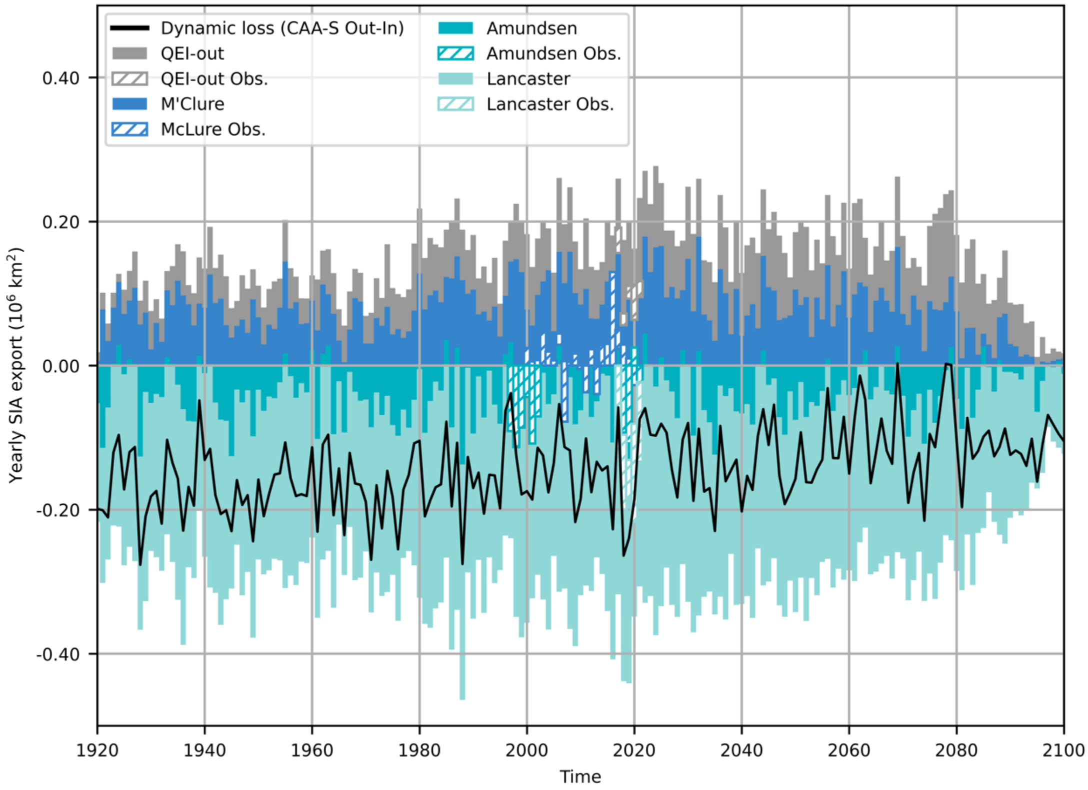

Fig. S3. Observed (hashed bars) and simulated (full bars) annual ensemble mean SIA fluxes in the CAA-S are defined positive when SIA enters the CAAS-S.

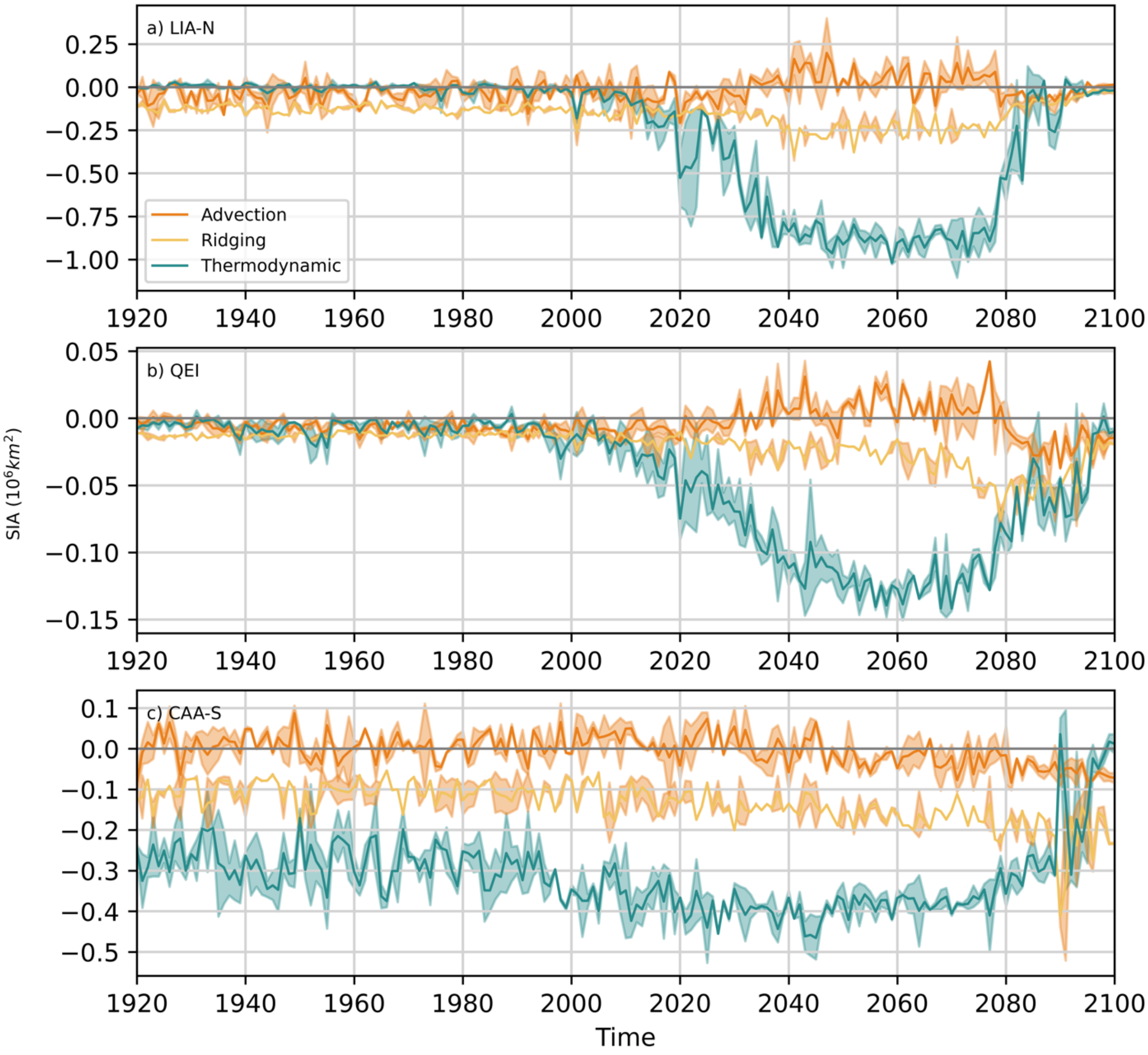

Fig. S4. Simulated ensemble mean and range dynamic (advection and ridging) and thermodynamic SIA loss integrated spatially and temporally over the melt season for the LIA-N (a), QEI (b) and CAA-S (c).

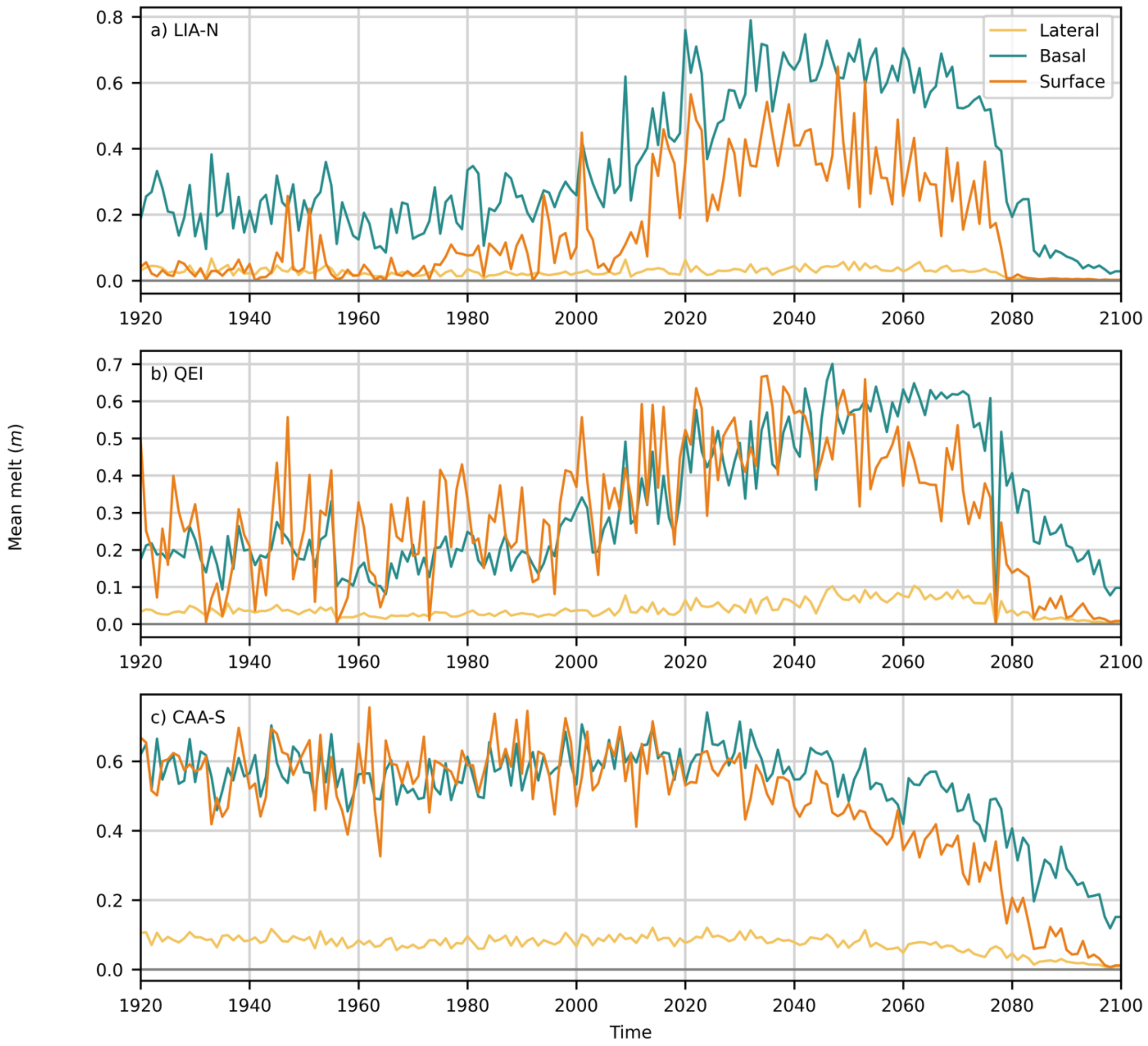

Fig. S5. Simulated mean lateral, basal and surface melt integrated spatially and temporally over the melt season for the LIA-N (a), QEI (b) and CAA-S (c) for ensemble member 1.

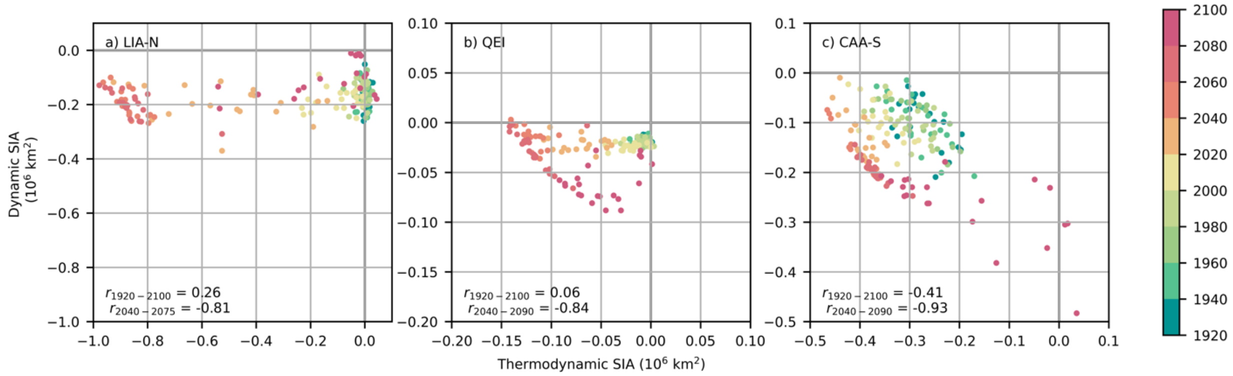

Fig. S6. Scatter plot of thermodynamic and dynamic SIA loss integrated over the melt season with linear correlation coefficients for the entire period (blue-red, 1920-2100) and the period after the drastic summer melting (orange-red, 2040-1990) for the LIA-N (a), QEI (b) and CAA-S (c). Each color covers a 20-year period from 1920 to 2100.

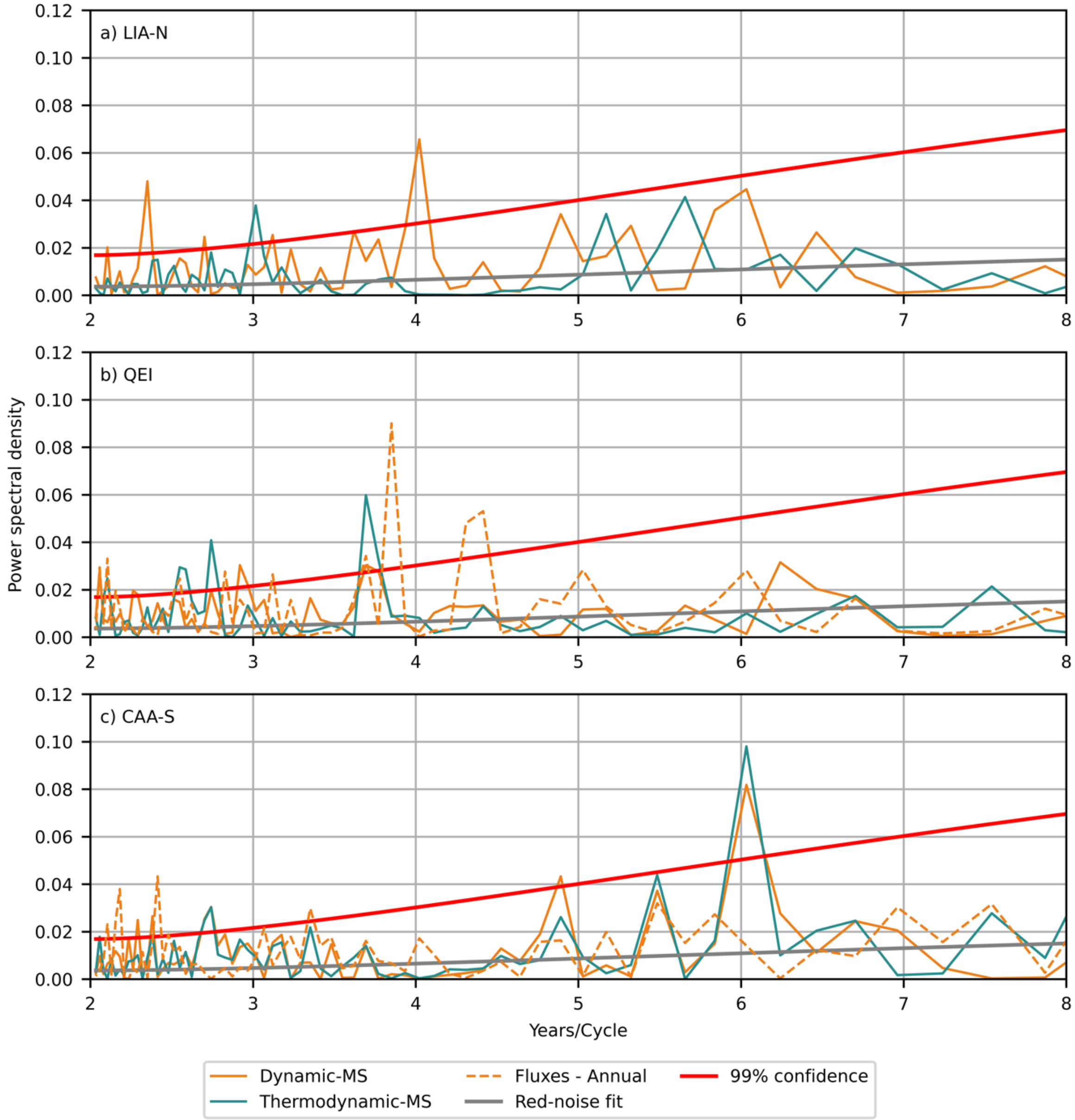

Fig. S7. Power spectral density distributions of the 20-years detrended thermodynamic and dynamic SIA loss from SIA tendencies integrated spatially and temporally over the melt season (MS) and annual SIA fluxes for the LIA-N (a), QEI (b) and CAA-S (c). Peaks are significant when surpassing the 99% confidence level (dotted red) against a null hypothesis that the data is red-noise.

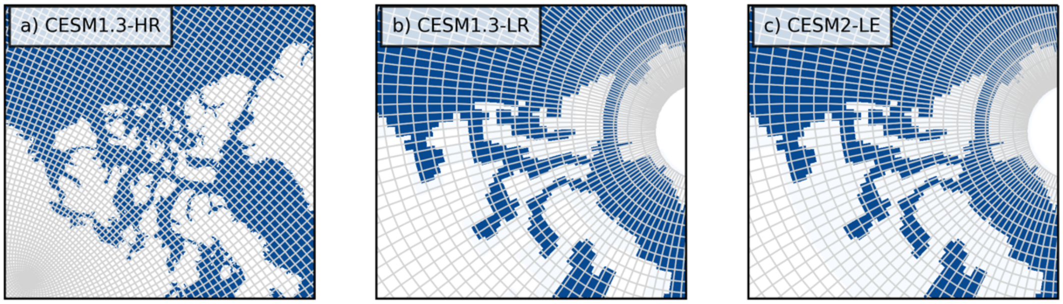

Fig. S8. Tripolar high-resolution CESM1.3-HR (a), and rotated pole lower resolution CESM1.3-LR (b) and CESM2-LE (c) grids of the Last Ice Area. The high and lower resolution grids are plotted every 12 and 2 grid points respectively for the sake of clarity.
